# Impact of ambidextrous leadership on innovative work behaviour and employee performance in the IT sector

**DOI:** 10.1016/j.heliyon.2024.e33124

**Published:** 2024-06-19

**Authors:** Mahendiren Dinesh Babu, Kushwaha Bijay Prasad, Upadhyaya Tara Prasad

**Affiliations:** a VIT Business School, Vellore Institute of Technology, Vellore, 632014, India; b Lumbini Banijya Campus, Tribhuvan University, Butwal, 32900, Nepal

**Keywords:** Ambidextrous leadership, Closing leadership, Employee performance, Innovative work behaviour, Opening leadership

## Abstract

The leadership role is crucial for motivating employees to do innovative work and discover new ideas and solutions. The IT sector is known for innovation. However, more studies are needed on the impact of ambidextrous leadership on innovative work behaviour (IWB) in the IT sector. This investigation explores the effects of ambidextrous leadership on innovative work behaviour and employee performance. This study employs a cross-sectional design, in which empirical data is gathered through scales adopted and modified from previous research. A total of 371 employees from IT companies participated in this survey. The selection of participants was carried out using a systematic sampling technique, with the survey being self-administered. In order to examine the structural relationships between opening and closing leadership, IWB, and employee performance, a method known as the partial least square structural equation modelling approach is used using SmartPLS 4.0 software. The findings of this study indicate that ambidextrous leadership has a significant and positive impact on IWB. Furthermore, it is revealed that IWB plays a significant role in influencing employee performance. Moreover, the impact of ambidextrous leadership, followed by innovative work behaviour, is more significant on employee performance. From a practical standpoint, this study offers valuable insights for IT organisations, which are renowned for their innovation but face a high attrition rate within the industry.

## 1 Introduction

In today's competitive world, an organisation's growth depends on its ability to develop and execute innovative ideas most effectively. Therefore, the organisation expects innovative work from its employees to sustain in the market. Innovative work behaviour encourages an individual to showcase their innovative ideas and thinking [1]. In addition, strong employee motivation for innovation can foster a climate that supports and encourages innovative working conditions [2,3]. Employees contribute innovative ideas and solutions when they believe they are essential to the organisation [4]. On the contrary, low employee motivation may reverse impact and inhibit innovative work behaviour [5,6].

The institution is exceedingly inventive, possessing proficient guidance and a propitious atmosphere for its workforce. Hence, leadership is crucial for motivating employees to work innovatively and discover new ideas and solutions [7]. In this regard, an ambidextrous leadership approach can positively motivate, encourage, and make employees feel they are significant contributors to organisational growth [8,9]. A leader with ambidextrous abilities can also foster flexibility within the organisation by balancing the requirement for consistency and effectiveness with the need for change and adaptation [10,11]. The term ambidextrous leadership refers to the ability of a leader to balance and integrate opposing or divergent tendencies to foster innovation and growth [10]. It involves simultaneously managing the present and the future, exploring new opportunities while optimising current operations [12]. An ambidextrous leader can create an environment of creativity and innovation throughout the organisation [13] by allocating resources to develop new ideas and encourage experimentation [9,14]. The two dimensions of ambidextrous leadership are leaders' opening and closing behaviour.

Each dimension of ambidextrous leadership is positively and significantly associated with work, organisational, and employee performance based on global studies' outcomes [15]. The literature shows that Closing Leadership Behaviour (CLB) significantly impacts innovative work behaviour and employee performance. CLB is defined as a leader's behaviour that will restrict variation in followers' behaviour through implementing corrective actions, establishing specific guidelines, and ensuring the achievement of goals. On the other hand, Opening Leadership Behaviour (OLB) encourages followers to explore novel concepts, experiment with alternates, increase independence, encourage taking risks and look at the status quo in the workplace [16]. OLB is inherently intended to provide more flexibility for employees to complete assigned tasks [10]. A leader taking part in OLB encourages subordinates to break out of these structures and accomplish tasks differently instead of continuing to act as they have in the past [17]. Since CLB has a clear vision of what to do and what not to do, the employees will follow the strict rules and do their work in their style and in an innovative way to achieve the goal of an organisation, which is set by their leader [18].

The role and style of leadership play a crucial role in the success of an organisation and the creative enhancement of its employees' productivity. While the existing literature has recognised this fact, it still needs to be improved in the context of the IT sector. The IT sector is renowned for its innovation, and the pace of such innovation determines the fate of organisations and their employees within this sector. However, more studies need to address the two approaches of ambidextrous leadership focusing on employee performance. Previous studies have predominantly concentrated on the Open Loop Behavior (OLB) approach, neglecting the Closed Loop Behavior (CLB) approach. Furthermore, some studies have employed a mixed leadership approach, combining OLB and CLB, but in different contexts, such as culture [19], organisational performance [20,21], organisational adaptability [22], personality effects [23], heterogeneity influence (Vishwakarma et al., 2020), and sectors such as the public sector [24,25] and Small-Medium Enterprises [26]. Thus, more research needs to be done to examine the impact of OLB and CLB on employee performance, specifically within the IT sector. Acknowledging the equal importance of both types of leadership, the present study aims to investigate the influence of CLB and OLB on employees' innovative behaviour and overall performance. Following the findings of [27], innovative behaviour may be developed as the combined ability of a team to create innovative and original ideas and then convert these ideas into action to enhance individual performance.

The study shall contribute by adding to the social exchange theory for leadership concerning employee performance in circumstances of innovative work behaviour. This research aims to enhance the prevailing collection of knowledge on leadership and innovation in organisations. It aims to offer scientific evidence that leaders who combine opening and closing behaviour are more successful in encouraging innovative behaviour, thereby positively impacting employee performance. This effect is observed to be independent of the leader's level of transformational leadership. Furthermore, this study identifies several potential research avenues in ambidextrous leadership. The findings of this research have theoretical and practical implications that may contribute to the advancement of leadership approaches and training processes. Specifically, these implications include the creation of new training modules that prioritise cultivating complementary leadership behaviour aimed at fostering employee performance.

## 2 Literature review

### 2.1 Theoretical underpinnings

An ambidextrous leadership is a leadership approach that motivates, encourages and makes employees feel they are significant contributors to organisational growth and creates conducive work environments for their associates [28]. The concept of an ‘ambidextrous organisation’ was initially introduced by Duncan (1976) and later adopted by Tushman and O'Reilly (2002) [29,30]. It refers to the challenge of effectively managing both the exploitation of existing competencies and the exploration of new fields of knowledge to succeed in organisational ambidexterity [29]. Tushman and O'Reilly (1996) argued that organisations must jointly utilise their current capabilities and explore new opportunities, necessitating a leadership style that can balance these two ostensibly opposing forces [15]. Ambidextrous Leadership has now been explicitly described for the process of innovation. Because of this, its innovative invention performance is enhanced compared to more traditional leadership models [17,31]. Ambidextrous leadership usually has two sub-concepts: opening and closing behaviour [32,33]. By balancing both behaviours, ambidextrous leaders can make an organisation's culture that can simultaneously improve and develop novel ideas, leading to long-term success and growth [3,34].

OLB has to do with discovery and coming up with new ideas. The behaviour includes pushing people to try new things, take risks, be creative, and look for new possibilities [35]. Leaders who show OLB are usually more at ease with doubt and uncertainty, and they can handle situations that are complicated and constantly changing [36]. [37] claim that this implies allowing employees the freedom to complete specific tasks. According to Ref. [17], Closing leadership behaviour prevents some organisations from achieving their goals. Here, a leader works to lessen workplace differences, intervenes often, establishes procedures and standards, and carefully monitors organisational objectives. Closing leaders only let their followers carry out duties in specified ways while closely supervised [17]. However, in both approaches, ambidextrous leadership ultimately changes employees' work environments, gives them the tools to do their jobs effectively, and makes them more motivated [38].

### 2.2 Innovative work behavior

Innovative work is job-related behaviour in which employees actively execute a new idea (West, 2002). These actions enhance the organisation's functioning [39]. The innovation process comprises two core stages: generation and implementation (Jiang et al., 2023b). The two stages of the creative process have different goals and may exhibit inconsistencies [40]. To tackle these contradictions effectively, the strong support of dynamic leaders is essential [41]. [39] found that ambidextrous leaders promote employees' innovative work behaviour [42,43]. These leaders encourage employees to display innovative behaviour by engaging in exploration and exploitation activities, thereby encouraging a thriving work environment [44]. The innovative effort of workers necessitates significant time and energy investment for the organisation's goal [43]. The correlation between leadership traits and employee creativity has consistently been a significant area of study in academic research, given the many environmental components that impact employee innovation performance [40,45].

### 2.3 Conceptual model

#### 2.3.1 Opening leadership behaviour on IWB

Opening leadership behaviour is a leadership style that fosters exploring new ideas, experimentation, and risk-taking [46]. When leaders demonstrate opening behaviour, they cultivate a sense of psychological security, encouraging employees to take risks and express novel ideas without apprehension of potential consequences [46,47] found that leaders exhibiting opening behaviour are likelier to promote innovative work behaviour among their employees. Additionally, previous studies have found a positive association between OLB and team innovation [11,48]. Further [13], study demonstrated that leaders who exemplify opening behaviour are more inclined to establish a climate that nurtures innovation within their organisations. However, while there is a relationship between opening leadership and innovative behaviour, other factors such as organisational culture, employee attitudes towards creativity and innovation, and the availability of resources that facilitate innovation may moderate this relationship [31]. Based on the argument above, the following hypothesis is proposed:

H1 Opening leadership positively impacts innovative work behaviour

#### 2.3.2 Opening leadership behavior on employee performance

Opening Leadership Behavior (OLB) can have a significant impact on the performance of employees. Leaders who display opening behaviours are generally more accessible and open to receiving input from their employees ([49]. This fosters a sense of engagement among employees, as they feel valued and empowered to contribute their thoughts and perspectives. A study by Rosing and Zacher (2023) [37] revealed that open leaders prioritise clear and transparent communication. This aids employees in comprehending their roles, objectives, and expectations more effectively, leading to improved performance outcomes. Furthermore, research findings from Ref. [50] have demonstrated that when leaders are receptive to input and ideas from their team members, they consider a more comprehensive range of perspectives in the problem-solving process. Opening behaviours, such as honesty, integrity, and transparency, contribute to the establishment of trust between leaders and employees. When employees trust their leaders, they are more likely to be motivated to perform at their utmost and align with the organisation's goals [51]. have discovered that leaders who exhibit opening behaviours often cultivate a positive work culture in which employees feel motivated and inspired to excel. Recognition, encouragement, and support from leaders can enhance morale and drive employees to perform at higher levels. Effective communication and a trust-based culture help prevent misunderstandings and conflicts within the team. When employees feel comfortable expressing their concerns or addressing issues openly, conflicts can be resolved more efficiently, thus minimising disruptions to performance [50]. Considering the points above, it is appropriate to put forward the following hypothesis:

H2 Opening leadership positively impacts employee performance

#### 2.3.3 Closing leadership behaviour on IWB

Closing leadership behaviour by leaders may impact employees' inclination to exhibit creativity in their job performance [36]. Leaders should adopt effective closing behaviour that engenders a sense of conclusion and focus on enhancing employees' enthusiasm, engagement, and commitment towards implementing innovative ideas [46]. Employees' enthusiasm to engage in creative work behaviour may be diminished if, for instance, a supervisor abruptly terminates exploratory endeavours without providing straightforward suggestions or encouragement for executing novel concepts [52]. Leaders must consider the influence of their closing leadership behaviour on employees' propensity to engage in creative problem-solving. Transitioning from exploration to exploitation necessitates leadership effectively communicating objectives and priorities, allocating resources, and offering guidance and support to personnel [53]. They can contribute to cultivating an environment that encourages and implements new ideas. Employees require more structure in promotion and execution than idea generation as they must critically evaluate ideas [54], organise and monitor their responsibilities and conduct, and effectively disseminate and reinforce their thoughts [53]. By the statements above, the subsequent hypothesis has been formulated:

H3 Closing leadership behaviour positively impacts innovative work behaviour

#### 2.3.4 Closing leadership behaviour (CLB) on employee performance

Closing leadership behaviour (CLB) pertains to the actions and conduct displayed by leaders that concentrate on completing tasks or projects, elucidating objectives, and ensuring the attainment of goals [55]. CLB aids in precisely defining goals and objectives, thereby furnishing employees with a distinct sense of purpose and comprehension regarding the expectations placed upon them. CLB emphasises the completion of tasks and efficacy, which can incentivise employees to concentrate on their respective duties and produce outcomes within predetermined timeframes [56]. Moreover, providing support and resources empowers employees to surmount challenges and impediments, culminating in enhanced performance [57]. CLB fosters a sense of accountability among employees by holding them accountable for their assigned tasks and resultant outcomes. Furthermore, CLB fosters employee collaboration and teamwork, enabling them to strive towards shared goals and objectives collectively. By highlighting the significance of collaborative efforts and utilising individual strengths, leaders cultivate a collaborative work environment where employees provide support and assistance to one another, ultimately augmenting overall performance [58]. CLB plays a pivotal role in influencing employee performance by promoting the clarity of goals, task orientation, accountability, feedback and support, and team collaboration. Leaders who embody effective CLB traits can positively impact employee performance and make valuable contributions to the organisation's overall success [59]. By the statements above, the following hypothesis is formulated:

H4 Closing leadership positively impacts employee performance

#### 2.3.5 IWB on employee performance

Innovative Work Behavior (IWB) has the potential to exert a considerable influence on employee performance across multiple dimensions [59]. Employees who manifest innovative work behaviour often possess a high proficiency in problem-solving [1]. They are adept at creatively addressing challenges, resulting in more efficient and effective solutions. This, in turn, has a positive impact on overall job performance. Furthermore [60], proposed that employees who engage in IWB are more inclined to embrace novel technologies, processes, and methodologies [60,61]. According to Ref. [62], this adaptability equips them to excel in work environments characterised by dynamism and constant evolution [63]. [63] indicates that employees who demonstrate creativity can generate original ideas, products, or processes that significantly augment performance by stimulating growth, productivity, and competitiveness. Innovative work behaviour can enhance employee motivation and engagement [41,63]. When employees feel empowered To contribute their ideas and witness the tangible effects of their innovative contributions, they are more likely to be motivated to operate at their optimal level [64].

Consequently, the cumulative impact of individual innovative work behaviour can significantly enhance organisational performance. When employees from different levels and departments engage in innovation, it fosters a culture that values creativity and continuous improvement, leading to more favourable outcomes for the organisation [65,66]. In line with the above statements, the following hypothesis is developed:

H5 Innovative Work Behaviour positively impacts Employee Performance

**Fig. 1:**
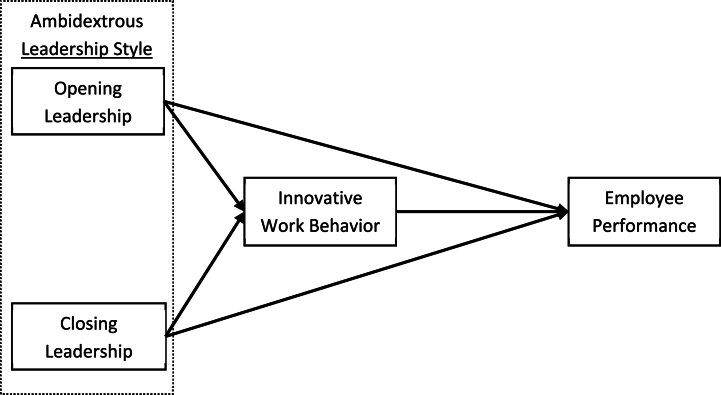
Conceptual model of ambidextrous leadership style.

The diagram in Fig. 1 illustrates the theoretical and speculative correlation between independent and dependent variables. It demonstrates the influence of opening and closing leadership behaviour on innovative work behaviour. Additionally, it indicates that both opening and closing leadership behaviour impact employee performance. Moreover, Fig. 1 indicates that innovative work behaviour is an intermediary in the relationship between opening leadership and employee performance and closing leadership and employee performance.

## 3 Research methods

### 3.1 Research design

This study aims to analyse the impact of ambidextrous leadership on firm performance. The IT sector in India has experienced a decline in performance for various reasons, including a recession caused by rapid technological changes. This has led to decreased revenue, profit, and overall performance. Adopting ambidextrous leadership is crucial to address this issue. This leadership style effectively balances exploration and exploitation activities, offering resistance against the adverse effects of the recession. Therefore, this study will examine the impact of ambidextrous leadership on firm performance in the IT sector.

This study is empirical, according to the Psychology research design categorisation system. This study used an association to examine the functional relationship of the three variables. Testing a mediation hypothesis makes this study explanatory. Additionally, this study used a latent variables design (LVD) or structural equation modelling (SEM), which has two parts: the inner model, which investigates the relationship between constructs, and the outer model, which investigates the relationship between indicators and constructs. The partial least squares method (PLS-SEM) was utilised to estimate SEM model parameters using variances. Purposive sampling was used to select participants [67].

### 3.2 Methods of data collecting and sample size

The researcher mobilised a cohort of IT employees to distribute 400 questionnaires. The primary objective was to ensure that adequate responses, surpassing 50 % of the total, were obtained to achieve the desired sample size. Predetermined statistical power analysis specified a minimum sample size. This minimal sample size ensures the statistical method's stability and model generalizability. G∗Power 3.1.9.7 software was used for analysis [68]. After analysis, two hundred ninety-three was the minimum suggested sample size. However, we collected data from three hundred seventy-one participants using the ten times rule [69]. Data collection took place between March and July of the year 2023. The data was gathered through a self-administered online questionnaire employing simple random sampling [69,70].

Before the primary data collection, a pilot study was conducted to ensure the reliability and validity of the questionnaire. Additionally, all ethical standards were adhered to, and respondents were assured of their anonymity, thereby establishing the credibility of the data. The survey instrument comprised two parts: the first part focused on the respondents' demographics, while the second part explored the constructs of the study utilising a five-point Likert scale. Despite lacking a comprehensive employee name database, their employee ID numbers were obtained through an additional procedure involving the collaboration of human resources managers across the organisation. The list of employees was acquired from the HR department, and systematic random sampling was applied, with every fifth employee selected based on their serial number. Furthermore, the distribution of questionnaires to each organisation was contingent upon the number of employees it had. For instance, organisations with a workforce of 300 individuals were provided with a total of 38 questionnaires, whereas companies with a staff size of 100 employees were allocated 13 questionnaires.

### 3.3 Measures

The present study used established measures evaluating opening leadership behaviour [71] and closing leadership behaviour [72] with seven components for each variable. The employee performance evaluation was conducted using a comprehensive measure of twenty-three items outlined by Ref. [72]. Previous research has proven the strength and accuracy of these instruments in many contexts [73,74]. The researchers in this study focus on the holistic style of leadership rather than the specific behaviour shown by leaders, which aligns with previous studies. Therefore, a unidimensional scale would be sufficient for comparing the two leadership philosophies. These are some examples of open and closed leadership styles.

### 3.4 Data analysis tools

The participants' responses were stored in the Google sheet. The same sheet was used for data analysis using SmartPLS 4.0 for structural framework testing, such as measurement model assessments, convergent validity, discriminant validity, and hypotheses testing [75] state that the Partial Least Squares (PLS) approach demonstrates effectiveness in predictive and explanatory research. The variance inflation factors (VIF) were derived and assessed in (Table 3) to evaluate the presence of multicollinearity and correlation among the variables of interest. By the multicollinearity criteria [76], this study did not encounter any issues with multicollinearity as the VIF was below 5.0, the tolerance was more significant than 0.2, and no association above 0.70 was observed among the variables. Data input and demographic analysis were executed employing SPSS version 27.0.

## 4 Findings and analysis

### 4.1 Respondents' profiles

Table 1 presents the demographic information of the survey participants. The participants of this study show that there is a good representation of all the socio-demographic categories. Fifty-two per cent of males and 48 per cent of females participated in this survey. Sixty-five per cent of participants were married, and 35 per cent were unmarried or single. Further, age-wise participation shows that 42 per cent were between 18 and 35, and 32 per cent fell between 36 and 50.

**Table 1:** Demographic profile of respondents.

**Gender**	**ƒ**	**%**	**Marital Status**	**ƒ**	**%**
Male	193	52	Single	130	35
Female	178	48	Married	241	65
***Total***	***371***	***100***	***Total***	***371***	***100***
**Age (Year)**	**ƒ**	**%**	**Qualifications**	**ƒ**	**%**
18–35	156	42	Diploma	48	13
36–50	119	32	Graduate	267	72
50+	96	26	Post-graduate	56	15
***Total***	***371***	***100***	***Total***	***371***	***100***
**Experience**	**ƒ**	**%**			
>5 Years	156	42			
6–10 Years	111	30			
10+ Years	104	28			
***Total***	***371***	***100***			

Further, 72 per cent of those respondents were undergraduates, 15 per cent were post-graduates, and the remaining were holders of diplomas. Twenty-eight per cent of participants have less than five years of work experience, 42 per cent of respondents had 6–10 years of job experience, while 30 per cent had a duration of 10 or more years. Additionally, 58 per cent of participants have over five years of experience in the organisation.

### 4.2 Measurement model assessment

The evaluation of the measurement model involves examining various statistical measures, such as outer loading analysis for individual items, VIF, composite reliability, extracted average variance (AVE), and the Heterotrait-Monotrait (HTMT) analysis (refer to Table 4). Furthermore, each construct satisfies the discriminant validity criteria, as shown in Table 4, where the correlation coefficient between latent variables is below 0.85, as Henseler et al. (2015) recommended.

Table 2 exhibits the statistical data about the outer loadings, Variance Inflation Factor (VIF), mean, and standard deviation. This tabular representation visually displays the significant loadings observed within the variable compared to those observed within other constructs. It becomes apparent through the examination of Table 2 that the outer loading of the items within all variables surpasses the critical threshold of 0.70. According to Hair et al. (2021), we have removed items EP6, EP14, EP19, and EP23 from the further analysis as their outer loading value is less than 0.70 [75]. This notable finding indicates that each retained item within the variables substantially contributes to measuring said variables.

**Table 2:** Assessment of measurement scale items.

Constructs	Codes	Factor Loading	V.I.F.	Mean	SD
Opening Leadership Behavior	OLB1	0.904	4.107	2.571	1.634
	OLB2	0.900	4.531	2.450	1.521
	OLB3	0.842	3.024	3.036	1.793
	OLB4	0.796	3.347	3.050	1.849
	OLB5	0.825	3.029	2.971	1.879
	OLB6	0.841	3.442	2.695	1.585
	OLB7	0.863	4.120	2.576	1.557
Closing Leadership Behavior	CLB1	0.754	1.695	2.883	1.752
	CLB2	0.755	1.865	3.369	1.833
	CLB3	0.838	2.431	2.981	1.572
	CLB4	0.754	1.816	3.307	1.816
	CLB5	0.816	2.188	2.940	1.832
	CLB6	0.838	2.309	2.614	1.587
Innovative Work Behavior	IWB1	0.729	2.498	4.400	1.920
	IWB2	0.710	3.104	4.848	1.827
	IWB3	0.835	3.278	4.329	1.886
	IWB4	0.809	3.561	4.224	1.963
	IWB5	0.875	4.649	3.421	1.979
	IWB6	0.846	4.352	3.195	2.102
	IWB7	0.822	2.985	3.771	1.872
	IWB8	0.843	4.501	3.436	2.172
	IWB9	0.827	3.918	3.536	2.146
	IWB10	0.852	3.538	3.860	1.998
Employee Performance	EP1	0.870	4.494	2.474	1.539
	EP2	0.798	4.331	2.767	1.567
	EP3	0.807	3.855	2.881	1.627
	EP4	0.865	4.546	2.502	1.600
	EP5	0.789	4.216	3.131	1.708
	EP6	*0.696*	3.701	3.519	1.697
	EP7	0.754	3.361	3.307	1.758
	EP8	0.826	3.749	2.650	1.676
	EP9	0.858	4.428	2.955	1.721
	EP10	0.818	3.611	2.424	1.588
	EP11	0.780	4.277	2.098	1.435
	EP12	0.769	4.358	2.179	1.499
	EP13	0.787	4.827	2.319	1.647
	EP14	*0.673*	2.261	2.907	1.784
	EP15	0.838	4.495	2.605	1.801
	EP16	0.732	3.499	3.362	1.830
	EP17	0.763	4.859	3.426	1.835
	EP18	0.799	4.974	2.933	1.811
	EP19	*0.658*	2.119	3.733	2.053
	EP20	0.823	4.896	2.948	1.685
	EP21	0.717	3.450	2.279	1.642
	EP22	0.757	4.200	2.226	1.674
	EP23	*0.688*	2.031	2.883	1.752

**Table 3:** Constructs' internal reliability and validity.

Constructs	Alpha	rho_A	CR	AVE	VIF	
					EP	IWB
Closing Leadership Behavior	0.882	0.887	0.910	0.629	2.154	2.048
Employee Performance (EP.)	0.970	0.971	0.972	0.607	–	–
Innovative Work Behavior (IWB.)	0.944	0.949	0.952	0.667	1.289	–
Opening Leadership Behavior	0.938	0.950	0.949	0.729	2.112	2.048

Furthermore, a meticulous investigation was conducted into multicollinearity among the items, employing the Variance Inflation Factor (VIF) as a diagnostic tool. This assessment revealed that all VIF values remained below the threshold of 5, suggesting the absence of multicollinearity within the outer model. Moreover, an evaluation of the centrality and variance of the responses was performed, employing the mean and standard deviation as measures. The range of mean values observed was near the average, indicating consistency and reliability within the responses. Furthermore, the standard deviation values observed suggest a relatively low deviation within the responses, thus implying high precision and accuracy.

### 4.3 Quality criteria assessment

Table 3 presents the reliability and validity of the inner model. Statistical measures such as Alpha, rho_A, Composite Reliability (CR), and VIF are employed to evaluate a scale or construct's internal consistency and reliability. Alpha is derived from Cronbach's Alpha, while rho_A is based on McDonald's omega coefficient. The results demonstrate that the Average Variance Extracted (AVE) exceeds 0.503, and the CRs are greater than 0.70. This indicates that the criteria for convergent validity have been met for all variables [77]. VIF is utilised to assess multicollinearity among predictors in regression analysis, with values below three considered good and five deemed acceptable. These findings suggest that the constructs in our study possess favourable internal consistency, as evidenced by high AVE values and a substantial amount of variance. Furthermore, the VIF values indicate the absence of significant multicollinearity issues among the predictors. Therefore, the measurement scales employed for the constructs can be deemed reliable, valid, and independent, essential for conducting accurate statistical analyses and drawing meaningful conclusions.

Table 4 provides an overview of the pairwise comparisons conducted to examine the various constructs. Table 4 explicitly showcases the Heterotrait-Monotrait (HTMT) ratio, which measures the correlations among all the variables utilised during this research. The HTMT ratio values observed in this study range from 0.337 to 0.859. It is generally recommended for the HTMT ratio values to be below 0.85. However, it is interesting to observe that two values in this study exceed the threshold of 0.85 but still need to reach the 0.90 mark. In line with the findings of [78], a variable with an HTMT ratio of up to 0.90 can still be considered acceptable. As a result, it can be concluded that the present study has successfully demonstrated discriminant validity among its reflective constructs, as highlighted by Ref. [79].

Table 5 provides a visual representation of the Fornell-Larcker Criterion Matrix, which showcases the variables utilised in the scope of this specific investigation. The Fornell-Larcker criterion, a widely recognised and esteemed method that enables the assessment of discrimination's validity, has been employed in this study. This exercise's primary objective is to compare the square root of the average variance extracted (AVE) and the correlation values associated with each construct present within the structural model, as [80] highlighted. Regarding each of the constructs thoroughly examined within the confines of this study, it is evident that the square root of the AVE values surpasses the values associated with the other constructs. By the principles outlined within the Fornell-Larcker Criterion, it is thus possible to determine that this study possesses discriminating validity, as discussed and elaborated upon by Ref. [81].

**Table 4:** HTMT ratio.

Constructs	CLB	EE	IWB	OLB
Closing Leadership Behavior				
Employee Performance	0.899			
Innovative Work Behavior	0.481	0.581		
Opening Leadership Behavior	0.768	0.857	0.427

**Table 5:** Fornell-Larcker criterion matrix.

Constructs	Closing Leadership Behavior	Employee Performance	Innovative Work Behavior	Opening Leadership Behavior
Closing Leadership Behavior	**0.893**			
Employee Performance	0.833	**0.879**		
Innovative Work Behavior	0.447	0.560	**0.817**	
Opening Leadership Behavior	0.715	0.868	0.429	**0.854**

### 4.4 Model fit assessment

The present study undertook an examination of the goodness-of-fit indices for the model. Specifically, the standardised root mean square residual (SRMR) was utilised for this purpose. The obtained SRMR value was 0.078, which falls below the maximum threshold of 0.08. This indicates that the model possesses good explanatory power, as [82] suggested. Moving on, the study of the significance of exogenous variables in the model was conducted by assessing effect size (f2) on endogenous constructs, measured in terms of r-square change. This approach is based on the methodology laid out by Ref. [83]. For the endogenous variable IWB, the f-The square value for CLB was found to be 0.052, while for OLB, it was 0.031. These values imply a large effect size for CLB and a medium effect size for OLB on IWB within the model. Similarly, when considering the endogenous variable of employee performance, the f-square values for CLB, IWB, and OLB were 0.521, 0.156, and 0.961, respectively. These results indicate a large effect size for CLB, IWB, and OLB on employee performance [84]. Lastly, the R-square values were examined, revealing that IWB possesses a weak explanatory power of 0.224, while employee performance demonstrates substantial explanatory power with a score of 0.866 [85].

### 4.5 Structural model assessment

**Fig. 2:**
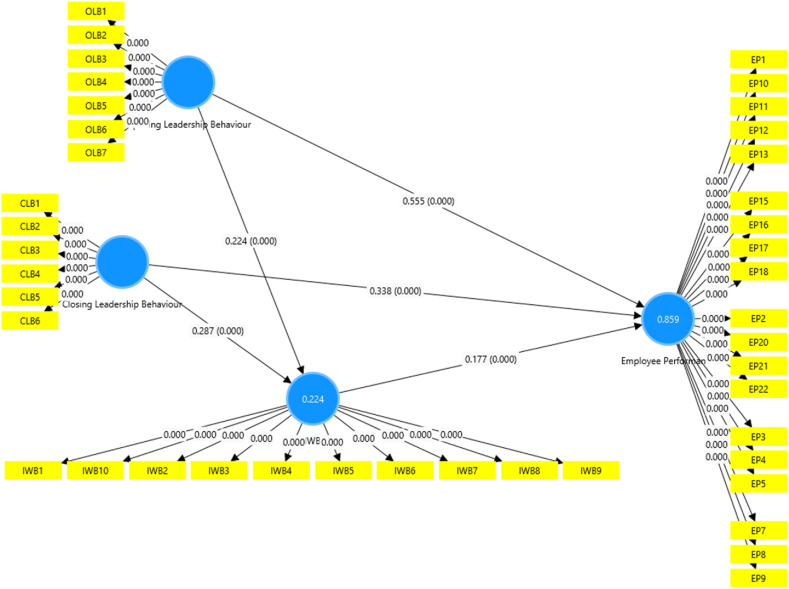
Path model.

To examine the hypotheses provided, the researcher utilised the bootstrapping technique, which involved generating 5,000 sub-samples from a population with a sample size of three hundred seventy-one. The r-square values, regression coefficient values, and p-values are displayed in Fig. 2. This technique was employed to obtain reliable and robust estimates of the parameters of interest. This approach is beneficial when the sample size is small, as it allows for assessing the sampling variability and constructing confidence intervals. The analysis results were then summarised in Table 6, Table 7, Table 8, which clearly illustrate the decision regarding the hypotheses. These tables provide an organised and concise representation of the findings, allowing for straightforward interpretation and comparison.

**Table 6:** Direct effects.

Hypotheses	β	Mean	SD	CI (2.5 %:97.5 %)	T Stat.	P Values	Decision
Closing Leadership Behaviour - > Employee Performance	0.338	0.338	0.028	[0.112, 0.335]	12.241	0.000	Accept
Closing Leadership Behaviour - > IWB	0.287	0.286	0.064	[0.463, 0.573]	4.467	0.000	Accept
IWB - > Employee Performance	0.177	0.177	0.019	[0.151, 0.410]	9.170	0.000	Accept
Opening Leadership Behaviour - > Employee Performance	0.555	0.555	0.029	[0.338, 0.439]	18.865	0.000	Accept
Opening Leadership Behaviour - > IWB	0.224	0.225	0.055	[0.127, 0.203]	4.050	0.000	Accept

**Table 7:** Indirect effects.

Hypotheses	β	Mean	SD	CI (2.5%–97.5 %)	T Stat.	P Values	Decision
Closing Leadership Behavior - > I.W.B. - > Employee Performance	0.051	0.051	0.013	[0.021, 0.061]	3.867	0.000	Accepted
Opening Leadership Behavior - > I.W.B. - > Employee Performance	0.040	0.040	0.010	[0.026, 0.078]	3.915	0.000	Accepted

**Table 8:** Total effect.

Hypotheses	β	Mean	SD	CI (2.5%–97.5 %)	T Stat.	P Values	Decision
Closing Leadership Behavior - > Employee Performance	0.388	0.388	0.029	[0.381,0.488]	13.366	0.000	Accepted
Closing Leadership Behavior - > IWB	0.287	0.286	0.064	[0.156,0.414]	4.467	0.000	Accepted
IWB - > Employee Performance	0.177	0.177	0.019	[0.126,0.202]	9.17	0.000	Accepted
Opening Leadership Behavior - > Employee Performance	0.594	0.595	0.03	[0.499,0.613]	19.735	0.000	Accepted
Opening Leadership Behavior - > IWB	0.224	0.225	0.055	[0.113,0.337]	4.05	0.000	Accepted

The researcher followed the methodology [86] outlined to understand the mediation process comprehensively. This methodology, widely recognised and accepted within the academic community, provides a systematic and structured approach to studying mediation. By adhering to this methodology, the researcher ensured the study's validity and rigour. One possible outcome of the mediation process is complete mediation. In this scenario, the direct impact between the independent and dependent variables lacks significance. However, the indirect effects, mediated through one or more intermediate variables, exhibit a significant relationship with the dependent variable. This indicates that the intermediate variables are crucial in explaining the relationship between the independent and dependent variables. The concept of complete mediation has been extensively discussed and elaborated upon by Ref. [86], who have provided valuable insights into its implications and interpretation.

In addition to complete mediation [86], also discuss the concept of partial mediation. This occurs when the direct and indirect effects between the independent and dependent variables are significant. In such cases, the intermediate variables are believed to explain the relationship between the two variables partially. It is important to note that partial mediation can manifest in different modes, including complementary and participating modes. These modes provide further nuance and depth to understanding the mediation process, highlighting the complexity and variability of the relationships under investigation. Using the bootstrapping technique and the comprehensive methodology outlined by Ref. [86] allowed the researcher to effectively examine the hypotheses and gain valuable insights into the mediation process. The findings in Table 6, Table 7, Table 8 provide a clear and concise overview of the decision regarding the hypotheses. Furthermore, the concepts of complete and partial mediation, elucidated by Ref. [86], shed light on how intermediate variables can influence the relationship between independent and dependent variables. Overall, this study contributes to the existing body of knowledge and enhances our understanding of the complex dynamics involved in mediation analysis.

This investigation has discovered a constructive direct impact of Closing Leadership Behavior on Employee Performance, with a beta coefficient of 0.338. A high degree of accuracy in estimating the genuine impact, a substantial t-statistic, and a negligible p-value indicate statistical significance. The proposition of a constructive direct impact is upheld, with a beta coefficient of 0.287 and a mean value of 0.286. Innovative Work Behavior (IWB) likewise demonstrates a constructive direct impact, bringing about an anticipated increase in employee performance. The acknowledged decision supports the constructive direct impact of IWB on Employee Performance. There are significant and positive impacts of opening leadership behaviour on IWB (β = 0.224, p < 0.001) and employee performance (β = 0.555, p < 0.001). Further, IWB (β = 0.177, p < 0.001) positively and significantly impacts employee performance.

The indirect effect reveals that innovative work behaviour is a positive mediator between a leader's opening and closing behaviour and the performance of their employees. The beta coefficient (β) of 0.051 signifies the positive indirect impact of closing leadership behaviour on employee performance through the mediating variable of innovative work behaviour (IWB). The mean value of 0.051 indicates that closing leadership behaviour influences employee performance, As Shown by its effect on IWB. The narrow confidence interval (CI) of [0.021, 0.061] indicates high precision in estimating the actual effect. The t-statistic of 3.867 and a p-value of 0.000 provide evidence of the statistical significance of the indirect effect. This study confirms that closing leadership behaviour positively influences employee performance through the mediating role of IWB. The beta coefficient of 0.040 demonstrates a positive indirect effect, with an average value of 0.040. The t-statistic of 3.916 and a p-value of 0.000 indicate that the indirect effect is statistically significant. The interplay between closing and opening leadership behaviour positively and indirectly impacts employee performance by influencing IWB. This suggests that employee participation in innovative behaviour partially mediates the relationship between leadership behaviour and employee performance. Cultivating an environment that fosters innovative work can strengthen the connection between leadership behaviour and employee performance.

Table 8 illustrates the comprehensive impact of independent variables on dependent variables. The beta value of 0.388 demonstrates a favourable association between closing leadership behaviour and employee performance, with a high level of accuracy in determining the actual effect. This effect may possess statistical significance, as indicated by the t statistic 13.366 and a p-value of 0.000. Additionally, a positive and significant relationship exists between employee performance and closing leadership behaviour. The beta coefficient of 0.287 suggests a direct positive effect of closing leadership behaviour on innovative work behaviour, with a beta value of 0.1777. The obtained results provide compelling evidence in support of the hypothesis that closing leadership behaviour has a positive impact on IWB. The narrow confidence interval of [0.156, 0.414] indicates a precise determination of the actual impact, with a t-statistic of 4.467 and a p-value of 0.000. Innovative behaviour at work and employee performance exhibits a positive correlation. The beta coefficient of 0.177 for IWB on employee performance signifies a direct positive effect. The average correlation between opening leadership behaviour and employee performance is 0.594, with high precision in forecasting the effect. A t-statistic of 19.735 and a p-value of 0.000 confirm the statistical significance of this finding. The outcome strongly supports the emerging relationship between opening leadership behaviour and IWB.

## 5 Discussion

The investigation aims to ascertain ambidextrous leadership's influence on innovative work behaviour and employee performance. The results show a positive and statistically significant correlation between opening leadership behaviour and employee performance. Our findings suggest that opening leader behaviour, which constitutes the primary aspect of ambidextrous leadership, fosters innovative behaviour (Zacher & Rosing, 2015). Opening leadership behaviour bestows power or authority upon subordinates to carry out individual tasks, thus motivating employees to approach their work creatively. Nevertheless, in certain instances, the delegation of tasks may yield unfavourable outcomes [87]. Moreover, opening leadership behaviour is deemed acceptable only when the employee's opening leader behaviour exerts a considerable influence on the level of creativity displayed by individuals in the workplace; however, organisations occasionally require a creative atmosphere. Opening leadership behaviour communicates to employees that errors made in the workplace can be advantageous as they serve as a basis for learning.

IWB plays a significant mediating role in enhancing the performance of employees. Hence, the presence of IWB is essential for this performance increase [88]. IWB, in this context, refers to the intentional process of introducing and implementing innovative and beneficial ideas within an organisation or workforce, aiming to improve its overall functioning [89]. Conversely, work denotes the expected level of performance that demonstrates how employees adapt to new changes, particularly in a dynamic environment. Ambidextrous Leadership involves being at the forefront of implementing and applying novel ideas proposed by employees [90]. This type of leadership signifies the ability to inspire and provide valuable insights, motivating employees to engage in innovative work behaviour [91]. Opening leadership behaviour supports colleagues in approaching tasks creatively, encourages them to experiment, allows subordinates to think and act independently, and supports their endeavours to challenge the current situation [46]. Therefore, opening behaviour positively influences both innovative work behaviour and employee performance. Following an uncertain situation, opening leadership behaviour directly impacts individual employee performance, regardless of the presence or absence of the mediating effect of IWB. The discovery of Leader-opening behaviour expands the range of follower behaviour by fostering creativity, encouraging independent thought and behaviour, and motivating followers to enhance the current scenario and predict employee behaviour (Karimi et al., 2023). This finding is consistent with prior research conducted by Refs. [2,20,91,92].

Moreover, it is essential to note that the Closing Leadership Behavior (CLB) concept has a considerable and notable influence on Innovative Work Behavior (IWB). Furthermore, it is also worth mentioning that CLB positively affects individual employees' performance. When we refer to closing leadership action, we are referring to the actions and behaviours demonstrated by a leader that aim to reduce the disparities and differences in the behaviours exhibited by their subordinates. This is achieved through implementing appropriate measures, establishing clear and concise guidelines, and evaluating the attainment of objectives [92] Our research findings indicate that closing leadership behaviour's impact on employee performance is marginally lower than opening leadership behaviour. However, it is essential to note that when innovative work behaviour acts as a mediator between closing leadership behaviour and employee performance, the impact of closing leadership behaviour becomes significantly higher. This is because well-defined objectives and the ultimate goal of the employee have a profound influence on their ability to accomplish tasks innovatively, consequently leading to enhanced individual employee performance. Therefore, the establishment of clear objectives and the cultivation of a work environment that encourages innovative approaches can significantly contribute to the improvement of individual employee performance.

Furthermore, when leaders clearly define objectives and goals that employees want and do not do, they will work with their innovative style to show their performance [93]. The prior study reveals that opening leadership behaviour influences employees' creative performance [50,92,94,95]. We predict that leader-closing behaviour will be connected with employee exploitation behaviour. Employees are more inclined to show behaviour that is compatible with their leaders' expectations and behaviours because they believe leaders are inspirations and have influence. As a result, leaders who are involved in specific closing behaviour, such as defining schedules, adhering to regulations and rules, establishing corrective measures, making guidelines, and monitoring the achievement of goals, should be more inclined to minimise variances in supporters' behaviour, which serves as the foundation for taking advantage activities. Few prior studies have investigated the negative correlation between employee innovative performance and a lack of opening and closing behaviour [88,96,97]. Finally, this study finds that implementing closing leadership behaviour, which includes establishing routines, monitoring goal achievement, and taking corrective actions, is expected to strengthen the Correlation between Closing Leadership Style and Employee Creative Performance. Prior research reveals that leaders' closing behaviour makes employees pay attention to the task [92,98]. When leaders exhibit closing behaviour, employees should depend on tried-and-true approaches rather than experimenting with new ways of functioning (Zacher & Rosing, 2015). Furthermore, employees ought to emphasise completing tasks efficiently and accurately. In turn, it is anticipated to lead to the development of creative and improved services and goods that support long-term progress in society, culture, and economic development.

## 6 Implications of the study

### 6.1 Theoretical implication

This study extends the context of Social Exchange Theory and Ambidextrous Leadership Theory by highlighting the significance of ambidextrous leadership in the IT sector, fostering innovative work behaviour and improving employee performance [1,90]. This could further refine existing leadership models and help researchers better understand the dynamics of leadership behaviour [95]. This study bridges the gap between organisational behaviour concepts such as ambidextrous leadership and innovative work behaviour, providing a deeper understanding of how these factors interact to influence employee performance [20,46,94]. It enriches the understanding of how leadership practices impact employee behaviour and organisational outcomes. In this study, we analyse the mediating effects of innovative work behaviour; the research offers insight into the underlying processes that explain how ambidextrous leadership influences employee performance. This emphasises how important it is for the organisation to cultivate an environment of creativity and innovation to achieve better outcomes.

### 6.2 Practical implication

This study signifies the factors that encourage employees to develop an IWB in the organisation. Employees who value their identities and goals more than the organisation and have a high level of interdependence in their self-construal are more likely to exhibit IWB when led by a transformational leader. Closing leadership is a mutual agreement between a leader and follower that focuses on defining subordinate expectations and offering rewards for performance. While excellent performance levels are achieved, closing leaders recognise their followers' requirements and meet their needs by rewarding them. Mutual benefits serve as the foundation of the relationship. Our research aimed to explore ambidexterity on the part of individual employees. This study analyses how closing and opening leadership promote creative work behaviour. These approaches allow organisations to determine whether opening and closing leadership influence one another. Further, it permits to differentiate between three distinct scenarios: (1) a balanced scenario, which describes a state in which both exploration and extraction operations are equally engaged or absent; (2) an unbalanced scenario, which describes a state in which the leadership of closing activities outweighs that of opening activities; and lastly (3) an unbalanced scenario, in which closing activities outweigh the leadership of opening activities. This study investigation has significant implications for professionals in the industry, researchers, scholars, and practitioners involved in the IT sector. The current research is foundational since it specifically examines the influence of an ambidextrous leadership style on employee performance in the IT sector.

## 7 Limitations and future direction

This study is limited to the IT sector but recommends further research to investigate other sectors with various forms of operations. This approach will provide a solid basis for interpreting findings and making decisions about ambidextrous leadership styles and employee performance. Second, the data was collected from the employees of companies in the southern region of India. Additionally, the current study evaluation is limited to the large IT sector companies only. Future research may consider start-up companies in this sector to have a more holistic view. The forthcoming studies may take a broader range of variables, such as gender disparities, to examine the impact of leadership styles on employee efficiency for a comprehensive investigation. Some elements that significantly enhance employee performance, including organisational culture, employee satisfaction, enthusiasm, and dedication, may be part of future studies. Therefore, additional research endeavours may be conducted to investigate the impact of these factors on employee performance comprehensively. Third, this study tested using social exchange and ambidextrous leadership theory. Only future studies may use the Conservational of Resources (COR) theory. Finally, this research used cross-sectional data to clarify relationships based on their theoretical basis, which may be another limitation of this study. It is advisable to use longitudinal data for future studies. Moreover, in existing academic research, there needs to be more specific information on the mediation effect of work engagement in the relationship between Ambidextrous leadership, perceived organisational support, and employee creativity. Therefore, it is recommended that future studies focus on conducting wide-ranging investigations to understand better the effects of Ambidextrous Leadership, perceived organisational support, and innovative behaviour on employee performance.

## 8 Conclusion

Closing leadership behaviour supports opportunity exploration and idea development. This is because favourable support from supervisors and monitoring enhances creativity and innovation. Closing leadership behaviour includes setting up routines, ensuring norms are observed, taking corrective actions, making rules, and keeping a close watch on how healthy goals are being fulfilled. This study is the first in India to assess the ambidextrous leadership theory in the context of innovative organisations, specifically within the information technology (IT) industry, at the individual employee level. The combined influence of opening and closing leadership behaviour significantly impacted innovation work behaviour. This effect was observed even after considering the positive individual effects of opening and transformational leadership behaviour. Specifically, the highest levels of team innovation were observed when both opening and closing leadership behaviour were exhibited at high levels. The opening and closing leadership behaviours are essential when comparing ambidextrous leadership at individual levels. Closing leader behaviour positively impacts innovative behaviour and individual employee success.

## Data availability statement

Data is available with the Corresponding author.

## CRediT authorship contribution statement

**Mahendiren Dinesh Babu:** Writing – original draft, Methodology, Formal analysis, Data curation, Conceptualization. **Kushwaha Bijay Prasad:** Writing – review & editing, Software, Methodology, Formal analysis. **Upadhyaya Tara Prasad:** Writing – review & editing, Supervision, Investigation.

## Declaration of competing interest

The authors declare that they have no known competing financial interests or personal relationships that could have appeared to influence the work reported in this paper.
